# Association of Psychiatric Illness or Psychotropic Medication Usage with Calcaneus Fracture

**DOI:** 10.7759/cureus.1977

**Published:** 2017-12-21

**Authors:** Cory F Janney, Jason T Goodrum, Daniel Jupiter, Cindy L Wigg, Kelly Carmichael

**Affiliations:** 1 Department of Orthopedics, United States Navy, University of Texas Medical Branch at Galveston; 2 Department of Orthopaedic Surgery and Rehabilitation, University of Texas Medical Branch at Galveston; 3 Preventive Medicine and Community Health, University of Texas Medical Branch; 4 Psychiatric and Behavioral Sciences, University of Texas Medical Branch; 5 Department of Orthopaedic Surgery and Rehabilitation, University of Texas Medical Branch

**Keywords:** psychiatric illness, calcaneus fracture, outcomes

## Abstract

Background

There is a noticeable lack of studies examining the connection between psychiatric illness and orthopaedic injury. The goal of this study is to determine if a relationship exists between calcaneus fracture and psychiatric illness or use of psychotropic medication.

Methods

A retrospective review was undertaken of calcaneus fracture patients at our institution from January 2011 through January 2014, and those with a diagnosis of psychiatric illness or history of psychotropic medication usage were identified. Medication records were analyzed along with medical histories taken during the initial encounter. If the patient was admitted, hospital notes for the hospitalization were reviewed to determine if any information was missed during the initial encounter. The date of injury, age, sex, insurance status at the time of initial encounter, psychiatric diagnoses or psychotropic medication use, and mechanism of injury were recorded. Any specific psychiatric diagnoses were collected from the patient charts, as was the presence of any specific prescribed psychotropic medications. After completion of the data collection, an attending psychiatrist verified the recorded data to ensure an accurate psychiatric assessment.

Results

A total of 85 calcaneus fractures met the inclusion criteria. In the population, there were 71 males and 14 females. The average age of the patients was 41.74 years, with 24% of patients having a diagnosis of psychiatric illness at the time of injury. The relative risk of a psychiatric illness in males compared to females was 0.31 (p = 0.009) while the relative risk of using psychotropic medication in males compared to females was 0.17 (p = 0.0007). Males were less likely to undergo operative intervention than females (p = 0.0001). The average age of a patient who either had a diagnosis or took medication for a psychiatric illness was 48.4 years, as compared to 39.7 years in those who did not (p = 0.014).

Conclusion

Males were less likely to have a psychiatric illness or be currently treated with psychotropic medications. A dedicated review of psychiatric history and prior medication may be useful during preoperative, perioperative, and postoperative treatment planning.

Level of Clinical Evidence: 4

## Introduction

Approximately 2% of all fractures are calcaneus fractures. Of these, approximately 60%-75% are intrarticular fractures with displacement. The majority of these fractures are in men, often working in an industrial field [1]. Displaced intraarticular calcaneus fractures are most often the result of high-energy trauma, such as a fall from height or a motor vehicle accident [1]. Operative intervention has shown better outcomes than nonoperative treatment [2]. Calcaneus fractures are often complex injuries, potentially involving severe damage to the soft tissue envelope producing severe swelling, skin blisters, compartment syndrome, skin necrosis, and open fractures [3]­.­ 

In 2014, it was estimated that 18.1% of all US adults had some form of mental illness [4]. Mental illness is defined as a mental, behavioral, or emotional disorder diagnosable within the previous year and of sufficient duration to meet the diagnostic criteria specified within the fourth edition of the Diagnostic and Statistical Manual of Mental Disorders (DSM-IV) [4]. Having a mental illness has been shown to be an independent risk factor for unintentional injury requiring hospital admission. The most common mechanisms of such injury were shown in one study to be falls and being hit by automobiles [5]. Individuals suffering from psychiatric illness have been shown to have abnormalities in perception and awareness of their surroundings. For example, schizophrenic patients have been shown to have a significantly increased pain tolerance [6-7]. Due to the fact that calcaneus fractures are commonly due to a high-energy trauma, it is possible that there is an association between calcaneus fractures and having a psychiatric illness or taking psychotropic medications.

There are no available studies on the association of calcaneus fractures with a diagnosis of psychiatric illness or utilization of psychotropic medication. The current research may guide future perioperative pain management protocols, lead to multidisciplinary treatment regimens, or indicate guided utilization of assistive services such as psychiatric resources and care management. 

## Materials and methods

A retrospective review of patients encountered at our institution with a diagnosis code for calcaneus fracture was performed. All research was performed with the approval of the institutional review board. All records were reviewed by an orthopaedic surgeon and an attending psychiatrist to ascertain proper diagnoses, surgical procedures, and psychiatric assessment and medications. Patients who presented to our institution between January 1, 2011, and January 1, 2014, with a diagnosis of a calcaneus fracture (ICD-9 code 825.0-825.2) of any severity and who were over 18 years of age were included in the study. Pregnant or incarcerated patients were excluded. The patients' medical records for the initial encounter regarding the injury were evaluated for a diagnosis of any reported psychiatric illness in the history and physical or consult documentation; any preexisting medical records, if available, prior to the encounter were reviewed. Medication records from the encounter and any timeframe available from prior to the encounter were analyzed for the utilization of psychotropic medications such antidepressants, antipsychotic medications, anxiolytics/hypnotics, stimulants, and mood stabilizers. 

Initial records provided by the institution included 120 separate patient encounters. These were evaluated to verify the diagnosis of calcaneus fracture and were excluded if the diagnosis was incorrect. After verification, 85 patients met the inclusion criteria. Date of injury, age, sex, operative intervention, insurance status at the time of the encounter, psychiatric diagnoses or psychotropic medication use, and mechanism of injury were collected. 

All variables (use of psychotropic drugs, whether a diagnosis of psychiatric illness had been made, whether either drugs were used or diagnoses made, insurance type, gender, suicide attempt, surgical intervention, high or low energy trauma, age, counts of diagnoses, and counts of medications) were summarized with means (standard deviations) or counts and proportions, as appropriate. The bivariate relationships between use of psychotropic drugs, whether a diagnosis of psychiatric illness had been made, or whether either drugs were used or diagnosis made, and the other variables were assessed with Fisher's exact test/chi-squared test or t-test, as appropriate. The bivariate relationships between counts of diagnoses or counts of medications were assessed using t-test or analysis of variance (ANOVA), and correlation, as appropriate.

## Results

From January 2011 through January 2014, 85 patients with calcaneus fractures presented to our institution. Of the total, 47% were self-pay patients; 22 underwent operative intervention at our institution. Of all patients received at our institution, two fractures were resultant from attempted suicide. Of the sample, 71 of the patients were male and the average age of all patients in the cohort was 41.74 years of age. The mean age of all patients with regards to medication usage, diagnosis, both medication and a defined diagnosis, and necessity of surgery is listed in Table 1. The data showed that 62% of the injuries were due to a low energy mechanism. In total, 24% of patients had a psychiatric diagnosis or were taking some sort of psychotropic medication; 15% of patients were only taking psychotropic medication; and 21% had an official psychiatric diagnosis.

**Table 1:** Association of Psychiatric Illness or Psychotropic Medication Use and Mean Age of the Patient at the Encounter

	Years	p-value
Medication usage	48.85	0.017
Official diagnosis	47.22	0.027
Medication usage or diagnosis	48.40	0.004
Patients undergoing operative intervention	45.68	0.050

The relative risk of having a diagnosis of a psychiatric illness in a male versus a female was 0.31 (p = 0.009) while that of being on medication was 0.17 (p = 0.0007), as seen in Table 2. The relative risk of having a diagnosis of a psychiatric illness or being on a psychotropic medication was 0.3 in males versus females (p = 0.003). Males were less likely to undergo operative intervention for calcaneus fractures than females (p = 0.0001). The relative risk of having a positive psychiatric diagnosis in those who attempted suicide compared to those who did not was 5.19 (p = 0.04). The relative risk of being on medication in those who attempted suicide compared to those who did not was 3.46 (p = 0.28). The relative risk of either was 4.61 (p = 0.05). The average age of a patient who either had a diagnosis or took medication for psychiatric illness was 48.4, as compared to 39.7 in those who did not (p = 0.014).

**Table 2:** Association of Psychiatric Illness or Psychotropic Medication Use With Patient Sex

		Relative Risk	Odds ratio	p-value
Sex (M vs. F)	Medication	0.17	0.09	0.00072
	Diagnosis	0.31	0.18	0.009
	Medication usage or diagnosis	0.3	0.15	0.003
	Surgery	0.24	0.08	0.0001

## Discussion

To date, there is very limited research with regards to psychiatric illness and its association with orthopaedic injuries. Psychiatric disorders can contribute to an intensified measure of the patient’s perceived pain and suffering, seemingly worsen disability, interfere with physical therapy, and contribute to noncompliance [8]. Catastrophic thinking, anxiety, posttraumatic stress disorder, and depression have been shown to be associated with a higher likelihood of taking opioid pain medication at one to two months after surgery for musculoskeletal trauma. This is without taking into account injury severity, fracture site, or treating surgeon [9]. Psychological stress has been shown to be a strong predictor of postoperative pain and analgesic consumption. Early identification of such a disorder has been shown to allow more effective intervention and better perioperative pain management [10]. As noted in the above results, 24% of patients had a psychiatric diagnosis or were taking some sort of psychotropic medication, which is slightly higher to the previously published average of 18.1%. 

Many patients with psychiatric illnesses take psychotropic medications. Between 2005-2008, 11% of Americans were prescribed antidepressant medications [11]. Selective serotonin reuptake inhibitors (SSRIs) are a very common medication prescribed for patients with depression. These medications are not without side effects. Sexual dysfunction, weight gain, altered sleep patterns, withdrawal symptoms, and loss of effectiveness are noted [12-13]. There is also the controversy as to whether SSRI medications may actually worsen suicidal thoughts and increase the risk of suicide [13]. Additionally, several studies have reported a reduction in bone mineral density in patients taking SSRIs for an extended period of time. A recent study of young, relatively healthy adults who had been taking an SSRI for at least three consecutive months demonstrated that these patients had a reduction of bone mineral density on calcaneal ultrasound [14].

This paper suggests that mental illness should be taken into consideration when deciding upon the best care for patients with calcaneus fractures. It supports the idea that a further medical history examination may be helpful in treating the patient not just for their immediate musculoskeletal needs, but also for their long-term mental considerations to yield the best possible outcome. Nearly a quarter of our patients had a diagnosis of psychiatric illness or were taking a psychotropic medication, but it is unclear if these considerations were taken into account when the patient was evaluated and treated.

There are several limitations to this paper. This is one of the first reviews attempting to establish a relationship between psychiatric illness and orthopaedic injuries. This could potentially lead to significant benefits in the outcomes of orthopaedic patients. As this was a retrospective review, there is a chance that patient diagnoses of psychiatric illness were not always included in the record if they were felt to be noncontributory to the encounter. If a review of the patient’s medications was not performed completely, these patients’ illness would be missed as well. Frequently, psychiatric history, in particular, is often not specifically discussed during the initial encounter. These inconsistently complete records were a limitation to this study, and also reflect an institutional limitation in patient history capture during evaluation. 

Another limitation may be selection bias, due to the frequent lack of follow-up with our patients. Our institution is located in a prime travel location. Many of the patients that we receive through our emergency department are vacationers who are injured while visiting, stabilized at our institution, and who follow-up when they travel back to their home. This potentially affected our sample size. The small sample size of the patients reviewed as well as the focused nature of the diagnosis may also make it more difficult to discover any associations. There were 85 patients that were included in the data analysis. Expanding the review to other types of fractures may yield data more generalizable to the population of patients we see. Due to the fact that this is a relatively small sample size, there may be sampling bias with regards to the number of males and females in the study. However, there does not appear to be any other studies like this to which we can compare.

Further research could include a prospective study with specific questions regarding the past psychiatric history of patients as well as a thorough medical reconciliation. The inclusion of other fracture types may provide more generalizability and may help determine if there is indeed an association, or if there is an association between particular fracture types and occurrence of psychiatric disorder.

## Conclusions

In our population of patients, males were found to have a higher likelihood of having a calcaneus fracture than females, while having a lower likelihood of having a psychiatric illness or using a psychotropic medication. A careful review of psychiatric illness may be helpful when approaching patients with calcaneus fractures as a psychological stress may affect outcomes including postoperative pain or analgesic consumption. 

Further research into the relationship between mental illness and fractures may help improve outcomes and patient satisfaction.
